# Cognitive Reserve, Incident Cancer, and Rate of Memory Decline in Later Life

**DOI:** 10.1093/geroni/igaa057.477

**Published:** 2020-12-16

**Authors:** Monica Ospina Romero, Willa Brenowitz, Eleanor Hayes-Larson, Sarah F Ackley, Elizabeth R Mayeda, M Maria Glymour, Lindsay Kobayashi

**Affiliations:** 1 University of California San Francisco, San Francisco, California, United States; 2 University of California, San Francisco, San Francisco, California, United States; 3 University of California Los Angeles, Los Angeles, California, United States; 4 University of Michigan, Ann Arbor, Michigan, United States

## Abstract

Cognitive reserve (cognitive skills and abilities acquired before onset of brain pathology) helps maintain cognitive function during aging. Cognitive decline after cancer treatment, known as “chemobrain,” is a prevalent outcome among older cancer survivors. It is unknown whether cognitive reserve buffers against acute neuropathological events such as cancer-related cognitive decline. We examined acute and long-term rate of memory decline associated with incident cancer diagnosis by education levels as proxy for cognitive reserve (low: <12 years; intermediate: 12 to <16 years; high: ≥16 years) in 14,449 adults aged 50+ in the US Health and Retirement Study from 1998-2016. Memory (z-scored) was assessed biennially as immediate and delayed word recall combined with proxy assessments. We used adjusted linear mixed models to determine long-term rates of memory decline before and after cancer diagnosis, and acute memory decline immediately after diagnosis (3,248 incident cases), and compared them with corresponding memory trajectories in cancer-free participants. Acute memory decline immediately after diagnosis was larger in those with low (-0.098 SD units, 95% CI: -0.150, -0.045) versus high (-0.038 SD units, 95% CI: -0.084, -0.008) education. Long-term memory decline after cancer was faster in those with low (-1.16 SD units/decade, 95% CI: -1.25, -1.07) versus high (-0.89 SD units/decade, 95% CI: -0.96, -0.82) education. Consistent with previous research showing an inverse cancer-dementia relationship, individuals with cancer had more favorable memory trajectories than cancer-free individuals with similar age and education. Among those with cancer, lower cognitive reserve was associated with greater acute and long-term memory decline after diagnosis.

